# Why Not?

**Published:** 1874-10

**Authors:** Chas. H. Evans


### WHY NOT?

BY CHAS. H. EVANS.

When a person calls on you having lost the lower molars, all or nearly all of the other inferior teeth being good, also having lost the upper anterior teeth, and still retains three or four good upper molars,—these may need some filling. A person in this condition generally thinks teeth are a nuisance and says, the quicker they are out the better; probably has not masticated solid food for years; been waiting intending to have a set some day; only wants teeth for looks, "can eat all they can get now."

Why not advise a lower partial plate fitted nicely to the remaining lower teeth, antagonising with the upper molars, so that the patient can eat with comfort. This is something that a person can appreciate at once, or, perhaps, after wearing them a week. On the contrary remove the upper molars in the above case with a view to replacing with a full upper set, and the person is maimed for life. The day has gone by, if it ever was, when artificial teeth are better than the natural ones. A man wearing upper and lower plates for years, said to me a few days since, "I would give five thousand dollars if I had or could obtain a good set of natural teeth." He knew how to prize them after they were lost forever.

But the person will say I can take the gas and lose these, and will not know it; I shall not have to pay for filling and I shall have to lose them sometime." A very convincing argument with many. Now, the dentist knows very well that if they are ordinarily good teeth, he can not replace them. Can he restore the round full outline of the cheeks? especially in the nervous temperaments. I will except some lymphatic ones. Can he prevent that premature old look, difficult to describe, but which every one has seen.

Why not then insert the lower partial plate? Perhaps you will say with Dr. Harris, see Dental Surgery, page 685. He says, "when there are but six teeth remaining in the inferior maxillary, it would be better to remove them and insert an entire dental apparatus, as it is exceedingly difficult and sometimes impossible to replace the others in such a way as to render them serviceable while the front part of the jaw is occupied with the natural teeth."

We will allow that this is good authority; that it is difficult sometimes very difficult, but not impossible. "When a man thinks he can and is determined, he will." His interest will increase with the number of difficulties to be overcome, and the satisfaction will be correspondingly great when it is accomplished.

Why not, if a person like the one here described, can be fitted successfully. We have the cast of the lower maxillary of an elderly person having of the natural teeth only two lower incisors remaining in place. These were sound, firm and stood together. A lower partial plate was fitted nicely, and worn with comfort. A full upper plate was also made at the same time. The latter is worn only occasionally, the patient being quite old. We have no doubt the lower plate would have been worn as little as the upper one, if the two incisors did not become disagreeably prominent when the lower plate was removed. For this reason it is retained and worn. These two teeth steady the lower plate very much, preventing side movement.

The dentist may think it is too much work, that it is not customary. In reply, we would say, if your patient is a reasonable person, he will very soon see that money is not the only consideration with you, and will have a little of that confidence in you which is so desirable to the successful practice of dentistry.

When they come back to you and say "you had ought to see me eat, how I did go without them so long I do not see." You will begin to receive your pay, it is similar to that which a painter feels when he looks and looks again at a painting of his creating, and is surprised and del ghted each time.

